# The cellular expression and proteolytic processing of the amyloid precursor protein is independent of TDP-43

**DOI:** 10.1042/BSR20200435

**Published:** 2020-04-28

**Authors:** David A. Hicks, Alys C. Jones, Stuart M. Pickering-Brown, Nigel M. Hooper

**Affiliations:** Division of Neuroscience and Experimental Psychology, School of Biological Sciences, Faculty of Biology, Medicine and Health, University of Manchester, Manchester Academic Health Science Centre, Manchester, M13 9PT, United Kingdom

**Keywords:** Alzheimers disease, Amyloid precursor protein, BACE1, Gene regulation, proteolysis, TDP-43

## Abstract

Alzheimer’s disease (AD) is a neurodegenerative condition, of which one of the cardinal pathological hallmarks is the extracellular accumulation of amyloid β (Aβ) peptides. These peptides are generated via proteolysis of the amyloid precursor protein (APP), in a manner dependent on the β-secretase, BACE1 and the multicomponent γ-secretase complex. Recent data also suggest a contributory role in AD of transactive response DNA binding protein 43 (TDP-43). There is little insight into a possible mechanism linking TDP-43 and APP processing. To this end, we used cultured human neuronal cells to investigate the ability of TDP-43 to interact with APP and modulate its proteolytic processing. Immunocytochemistry showed TDP-43 to be spatially segregated from both the extranuclear APP holoprotein and its nuclear C-terminal fragment. The latter (APP intracellular domain) was shown to predominantly localise to nucleoli, from which TDP-43 was excluded. Furthermore, neither overexpression of each of the APP isoforms nor siRNA-mediated knockdown of APP had any effect on TDP-43 expression. Doxycycline-stimulated overexpression of TDP-43 was explored in an inducible cell line. Overexpression of TDP-43 had no effect on expression of the APP holoprotein, nor any of the key proteins involved in its proteolysis. Furthermore, increased TDP-43 expression had no effect on BACE1 enzymatic activity or immunoreactivity of Aβ_1-40_, Aβ_1-42_ or the Aβ_1-40_:Aβ_1-42_ ratio. Also, siRNA-mediated knockdown of TDP-43 had no effect on BACE1 immunoreactivity. Taken together, these data indicate that TDP-43 function and/or dysfunction in AD is likely independent from dysregulation of APP expression and proteolytic processing and Aβ generation.

## Introduction

Alzheimer’s disease (AD) is a progressive neurodegenerative disease and the most common form of dementia [1]. It is traditionally characterised by a bipartite pathology featuring plaques comprising amyloid β (Aβ) peptide and neurofibrillary tangles containing the microtubule-associated protein τ [1,2]. The former is produced by sequential proteolytic cleavage of the amyloid precursor protein (APP), of which there are three major isoforms (APP_695_, APP_751_ and APP_770_) [3,4].

There are two predominant proteolytic pathways of APP processing, though other pathways have been identified [5]. In neurons, amyloidogenic processing is the minor pathway and involves sequential cleavage by β-secretase, BACE1 and the γ-secretase complex (comprising presenilin 1 or 2, nicastrin, Aph1 and Pen2) [6–8]. This ultimately releases Aβ species (Aβ_1-40_ and Aβ_1-42_ are the most significant), which may play a role in the pathogenesis of AD [9]. The second, non-amyloidogenic, pathway involves α-secretase cleavage of APP, a function ascribed to the metalloprotease ADAM10 in neurons [10]. This cleavage occurs within the Aβ region [11,12], precluding generation and deposition of Aβ. A large, soluble, N-terminal ectodomain, the neuroprotective sAPPα, is shed from the cell surface as a product of this pathway [13]. The most C-terminal region of the APP holoprotein is referred to as the amyloid precursor protein intracellular domain (AICD), through which a number of signalling pathways and transcriptional regulatory events are mediated [14–17].

There is an emerging view that transactive response DNA binding protein 43 (TDP-43) plays a key role in AD pathogenesis. TDP-43 was first linked to neurodegenerative disease through its discovery as a component of classical ubiquitination inclusions in motor neuron disease and frontotemporal dementia [18]. Since then, several recent findings have indicated a key role in AD [19–21]. TDP-43 is a predominantly nuclear protein, although it does shuttle between the nucleus and cytosol [22]. It functions as a nucleic acid binding protein, regulating in excess of 6000 targets. It can modulate all aspects of RNA regulation: processing, splicing, transport and translation, targeting both coding and non-coding RNAs [23].

TDP-43 inclusions have been identified in AD brains in the form of high molecular weight [24] and phosphorylated [25] TDP-43 species. This has been subsequently validated by several neuropathological studies and developed into a staging scheme for TDP-43 pathology in AD [26–30]. In support for an active role for TDP-43 in AD progression, the presence of TDP-43 inclusions in AD is associated with a higher Braak stage and greater impairment in cognitive performance [20,21,31]. It is considered unlikely that there are any significant TDP-43 mutations in AD cases [32].

However, despite evidence showing the presence of TDP-43 inclusions in AD cases and an inverse correlation with cognitive performance, mechanistic insight is sparse. It is neither clear why TDP-43 forms inclusions in neurodegenerative disease generally, nor what specific effects this might have in AD. Overexpression of TDP-43 in AD transgenic mice has been shown to reduce Aβ plaque number, through mechanisms unclear [33], although TDP-43 depletion increased Aβ uptake by microglia [34], which corresponds to the decreased plaque formation in a mouse model [35]. In addition, TDP-43 regulates τ splicing [36] and suppresses its expression [37]. TDP-43 may modulate mitochondrial function [38,39], as evidenced by a recent report of small molecule inhibition of TDP-43 localisation to mitochondria having beneficial effects in an AD transgenic mouse [40].

Despite the centrality of APP, its proteolytic processing and metabolites in AD, there has only been one investigation, in mice, of the ability of TDP-43 to modulate APP processing [41]. To this end, we sought to elucidate the role of TDP-43 in modulating the expression and proteolytic processing of APP in human cells. The aim of the present study was to describe, for the first time, the possible dependence of APP processing on TDP-43 and the effects of possible dysfunction of this axis in the context of AD.

## Experimental procedures

### Materials

All chemicals were purchased from Fisher Scientific (Loughborough, Leicestershire, U.K.) unless otherwise stated. Actinomycin D was from Sigma–Aldrich (Gillingham, Dorset, U.K.).

Primary antibodies used: for APP (Y188; Abcam, Cambridge, U.K.; RRID:AB_2289606 and 22C11; Merck Millipore, Watford, Hertfordshire, U.K.; AB_94882), TDP-43 (Proteintech, Manchester, U.K.; AB_ 2200520), AICD (BioLegend, London, U.K.; AB_2564761, raised against a neo-epitope around the VMLK sequence at the ε-cleavage site), Tip60 (Novus Biologicals, Abingdon, Oxfordshire, U.K.; AB_1199339), Fe65 (Novus Biologicals; AB_789863), FLAG (Sigma–Aldrich; AB_439687), ADAM10 (Proteintech, Cat #25900-1-AP), BACE1 (Cell Signaling Technology, Leiden, The Netherlands; AB_1903900), nicastrin (Cell Signaling Technology; AB_10694688), Pen2 (ABcloncal, Woburn, MA, U.S.A.; AB_2771856), PS1 (Abcam; AB_1310605), PS2 (Cell Signaling Technology; AB_10831052), fibrillarin (Abcam; AB_1523617) and GAPDH (Proteintech, AB_2107436).

### Methods

#### Cell culture

SH-SY5Y cells (RRID:CVCL_0019) were cultured in 1:1 Dulbecco’s modified Eagle’s medium: Ham’s F12 with UltraGlutamine (DMEM F12, Cat #BE04-687F/U1, Lonza, Slough, Berkshire, U.K.), supplemented with 10% foetal bovine serum (FBS) (Life Technologies, Thermo Fisher Scientific, Paisley, U.K.), 100 U/ml penicillin, 100 µg/ml streptomycin (both Life Technologies) and 1% non-essential amino acids (Sigma–Aldrich). Cells were incubated at 37°C and 5% CO_2_ in a humidified atmosphere. For differentiation, cells were seeded and cultured in full growth medium (above) for 24 h, then differentiated for 7 days in DMEM F12 supplemented with 1% FBS (Cat #631107, Takara, Saint-Germain-en-Laye, France), 100 U/ml penicillin, 100 µg/ml streptomycin, 1% non-essential amino acids and 1 µM retinoic acid. SH-SY5Y cells overexpressing APP isoforms were generated as previously described [42].

The iPSC line, OX1-19 (obtained from S. Cowley, University of Oxford) was maintained on Matrigel (BD Biosciences, Wokingham, Berkshire, U.K.) in mTeSR1 medium (StemCell Technologies, Cambridge, U.K.) containing 50 U/ml penicillin and 50 µg/ml streptomycin (Life Technologies) in a humidified incubator at 37°C in a 5% CO_2_, 95% air atmosphere. iPSC pluripotency and successful cortical neuron differentiation were confirmed using immunofluorescence microscopy with appropriate markers [43]. The iPSCs were differentiated into cortical neurons as described previously [44,45], using dual-SMAD inhibition by 1 µM dorsomorphin and 10 mM SB431452 (Bio-Techne, Abingdon, Oxfordshire, U.K.). Following successful differentiation, neural progenitor cells (NPCs) were re-plated on day 35 post-induction at 300000 cells/well on to poly-ornithine and laminin-coated (Sigma–Aldrich) six-well tissue culture plates and cultured until between days 75 and 90 post-induction with media changes every 2–3 days. Post-induction culture medium was 1:1 DMEM F12: neurobasal medium containing B27 and N2 supplements, 2 mM l-glutamine, 100 µM 2-mercaptoethanol, 25 µM insulin, 100 U/ml penicillin, 100 µg/ml streptomycin (all Life Technologies) and 0.5% non-essential amino acids.

#### Inducible cell line

For the generation of the inducible cell line, the TRE-x system was used (Thermo Fisher Scientfic). Two plasmid vectors were used: the regulatory pcDNA6/TR and pT-Rex-DEST30 (expressing FLAG-TDP-43) (GeneArt, Thermo Fisher Scientific). Both vectors were transfected into SH-SY5Y cells (2 µg total DNA, 6:1 ratio) using the Amaxa 4D-Nucleofector on programme CA-137 (Lonza). After 48 h, cells stably expressing both plasmids were selected using blasticidin (5 µg/ml) and G418 (500 µg/ml), selecting for the regulatory and expression vectors, respectively. TDP-43 expression was induced using 500 ng/ml doxyxlycline hyclate (Sigma–Aldrich) for 72 h. Expression of FLAG-TDP-43 was verified using immunoblotting.

#### siRNA knockdown

SH-SY5Y cells were differentiated for 7 days as described and then nucleofected with siRNA duplexes targeting either APP (100 nM; Ambion Silencer Select, Thermo Fisher Scientific; ID s229520, siRNA #1 or Dharmacon ON-TARGETplus SMARTpool, Horizon Discovery Cambridge, U.K.; Cat #L-003731-00-005, siRNA #2), TDP-43 (120 nM; Ambion Silencer Select, Thermo Fisher Scientific; ID s23879, siRNA #1 or Dharmacon ON-TARGETplus SMARTpool, Horizon Discovery Cambridge, U.K.; Cat #L-012394-00-005, siRNA #2) or a non-targeting control (Negative Control siRNA; Qiagen, Manchester, U.K.) according to the manufacturer’s instructions (Amaxa 4D-Nucleofector; programme CA-137). Cells were further cultured for 72 h and target knockdown verified using immunoblotting.

#### Immunocytochemistry

Cells were cultured on gelatin-coated glass coverslips (BioReagent, Sigma–Aldrich). After treatment, cells were fixed in 4% paraformaldehyde for 10 min then washed in phosphate-buffered saline (PBS). Cells were subsequently permeabilised in 0.5% Triton X-100, washed in PBS and incubated with blocking buffer (0.5% fish skin gelatin (FSG, Sigma–Aldrich) in PBS) for 1 h. Coverslips were incubated with primary antibody for 1 h (in 0.5% FSG, 0.5% Triton X-100 in PBS), washed in PBS and incubated for 1 h in secondary antibody (0.5% FSG, 0.5% Triton X-100 in PBS and either Alexa Fluor 488 (Thermo Fisher Scientific; AB_2610666), Alexa Fluor 555 (Abcam; AB_2801638) or CF 647 (Sigma–Aldrich, Cat #SAB4600183). After washing, coverslips were incubated with 1 µg/ml DAPI (emp Biotech, Berlin, Germany) for 5 min and mounted on microscope slides with Prolong Diamond mounting medium (Thermo Fisher Scientific). Images were acquired on an Olympus IX83 inverted microscope using Lumencor LED excitation, a 40× or 60× objective and the Sedat QUAD filter set (Cat #89000, Chroma Technology, Bellows Falls, VT, U.S.A.). The images were collected using a Retiga R6 Q-Imaging CCD camera and Metamorph v7.8.4.0 (Molecular Devices, San Jose, CA, U.S.A.). Images were then processed and analysed using ImageJ (NIH, U.S.A.).

#### Cell lysis

Cells were washed twice in ice-cold PBS and harvested in PBS. Cells were pelleted at 3000×***g*** for 5 min (4°C) and re-suspended in 6× volume of lysis buffer (RIPA buffer: 50 mM Tris/HCl (pH 8.0), 150 mM sodium chloride, 1% Igepal CA-630 (Sigma–Aldrich), 0.5% sodium deoxycholate, 0.1% SDS, 1 mM sodium fluoride, 1 mM sodium orthovanadate, and Complete Protease Inhibitor cocktail (Roche Diagnostics, Burgess Hill, West Sussex, U.K.)). Lysis was performed for 30 min on ice, followed by centrifugation at 3000×***g*** for 5 min (4°C) to yield the RIPA-soluble fraction as the supernatant, which was used for immunoblotting.

#### Determination of protein concentration

Protein concentration in the RIPA-soluble fraction was determined using the bicinchoninic acid (BCA) method [46], using a Pierce BCA Protein Assay Kit (Thermo Fisher Scientific). Absorbance at 562 nm was measured using a plate reader (ELx800, BioTek, Swindon, U.K.). Sample concentration was determined using bovine serum albumin (BSA) as a standard at concentrations from 0 to 1 mg/ml.

#### SDS/PAGE and immunoblotting

Protein samples were separated by electrophoresis at 120 V for 90 min on a polyacrylamide gel. After SDS/PAGE, proteins were transferred to polyvinylidene fluoride (PVDF) membranes (Bio-Rad, Hemel Hempstead, Hertfordshire, U.K.). Blots were incubated for 2 h in blocking solution (5% (w/v) milk power, 2% (w/v) BSA in TBS + 1% (v/v) Tween-20 (TBST)). The blots were then incubated overnight in primary antibody (5% (w/v) milk powder in TBS). Blots were washed 4 × 10 min with TBST before the addition of secondary antibody (HRP–conjugated anti-IgG; 5% (w/v) milk powder in TBST, 1:5000 (Thermo Fisher Scientific)) for 1 h, followed by 4 × 10 min washes with TBST. Protein bands were visualised by chemiluminescence (Clarity Western ECL Blotting Substrate, Bio-Rad) using a G:BOX and GeneTools software (Syngene, Cambridge, U.K.).

#### Quantitative PCR

RNA was isolated from differentiated SH-SY5Y cells using the RNeasy Mini Kit according to the manufacturers’ instructions (Qiagen). cDNA was subsequently prepared using the Applied Biosystems High Capacity cDNA Synthesis Kit after which quantitative PCRs (qPCRs) were prepared as follows (total 20 µl): 1 µl cDNA, 500 nM each of forward and reverse primers with PowerUp SYBR Green Master Mix (Thermo Fisher Scientific). Thermal cycler (QuantStudio 3, Applied Biosystems, Thermo Fisher Scientific) parameters were set as follows: 2 min @ 50°C, 2 min @ 95°C and 40 cycles of 15s @ 95°C, 15s @ 53°C and 60s @ 72°C and data analysed using the ΔΔ*C*_T_ method with succinate dehydrogenase complex, subunit A (SDHA) as a reference gene. Primer sequences were as follows: BACE1 (F: GTAAAGCAGACCCACGTTCC; R: CAATGATCATGCTCCCTCCG) and SDHA (F: CAGCATGCAGAAGTCAATGC; R: ACGTCTTCAGGTGCTTTAGG).

#### BACE1 activity assay

Differentiated SH-SY5Y cells were lysed in assay buffer (10 mM sodium acetate, 1.5 mM NaCl, 0.1% Triton X-100, 0.32 M sucrose, pH 5.0) for 30 min on ice, before clarification by centrifugation (5000×***g***, 5 min) and protein quantitation of the supernatant. Subsequently, 2 µl of BACE1 substrate (Abcam) was added to 50 µl supernatant (containing 50 µg total protein) followed by kinetic output measurement on a fluorescent microplate reader at Ex/Em = 335/495 nm (Synergy HT, Biotek). Rate (ΔRFU) was calculated in the linear reaction phase.

#### Amyloid-β enzyme-linked immunosorbent assay

Conditioned media samples (48 h) were isolated from differentiated SH-SY5Y cells and centrifuged at low speed to remove cell debris (500×***g***, 5 min). The resultant supernatant was concentrated ten-fold using a Vivaspin 2 (2000 MWCO) and the protein concentration assessed. Conditioned media samples were then added to enzyme-linked immunosorbent assay (ELISA) plates to quantify Aβ_1-40_ (5 µg total protein, Cat #KHB3481) and Aβ_1-42_ (30 µg total protein, Cat #KHB3544) (both Thermo Fisher) ELISAs were performed according to the manufacturer’s instructions, including incubation of condition medium with biotinylated detection antibody, followed by HRP–conjugated secondary antibodies, chromagen and finally the stop solution, resulting in a colorimetric response proportional to target abundance, the absorbance of which was measured on a microplate reader at 450 nm (ELx800, Biotek).

### Statistical analysis

All experiments are *n*=3 unless otherwise indicated, where *n* indicates independent experiments on independent cell cultures. Statistical tests were either Mann–Whitney U test, Student’s *t* test (ELISA and BACE1 activity data only) or Kruskal–Wallis with Dunn’s *post hoc* test as indicated; *P*<0.05 (*), *P*<0.01 (**), *P*<0.001 (***) or *P*<0.0001 (****). Error bars indicate standard deviation. All statistical analyses were performed using GraphPad Prism 8 (GraphPad Software, Inc., La Jolla, CA, U.S.A.).

## Results and discussion

### APP and TDP-43 have distinct intracellular locations in cultured neuronal cells

**Figure 1:**
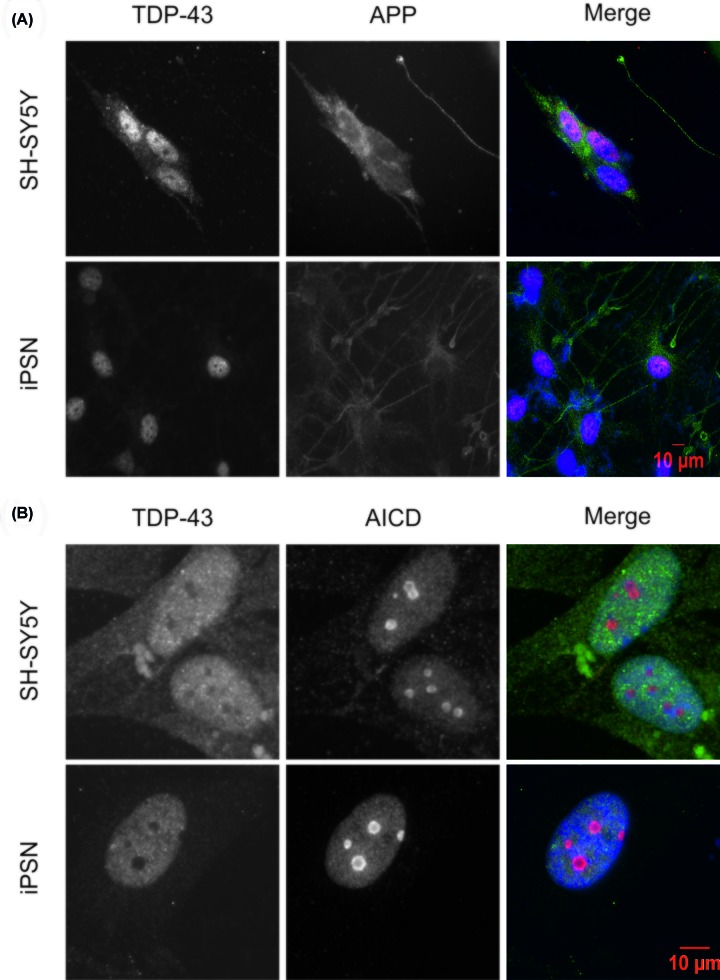
TDP-43 does not co-localise with either the APP holoprotein or its intracellular domain SH-SY5Y or OX1-19 iPSCs were cultured and differentiated as described, followed by fixation and immunocytochemistry using primary antibodies against TDP-43 and (**A**) APP holoprotein (antibody 22C11) and (**B**) AICD. iPSN denotes iPSC-derived neurons.

In order to investigate a possible direct relationship between APP and TDP-43, putative co-localisation was assessed using immunofluorescence microscopy. Using differentiated SH-SY5Y cells and, separately, OX1-19 iPSC-derived neurons (iPSNs), the localisation of the APP holoprotein and TDP-43 was investigated. As expected, TDP-43 was localised exclusively in the nucleus, whereas the APP holoprotein was excluded from the nucleus (Figure 1A). AICD translocates to the nucleus after proteolysis of the APP holoprotein [15,17]. Using an AICD-specific antibody (targeting a neo-epitope) [47], we probed the subcellular localisation of AICD in comparison with TDP-43. Though there was some evidence of AICD immunoreactivity throughout the cell, AICD was most strongly localised to the nucleus. More specifically, AICD was present as a component of several large subnuclear structures. In contrast, TDP-43 was completely excluded from these structures, seen as voids in the TDP-43 immunostaining. This was recapitulated in SH-SY5Y cells and iPSNs (Figure 1B).

**Figure 2:**
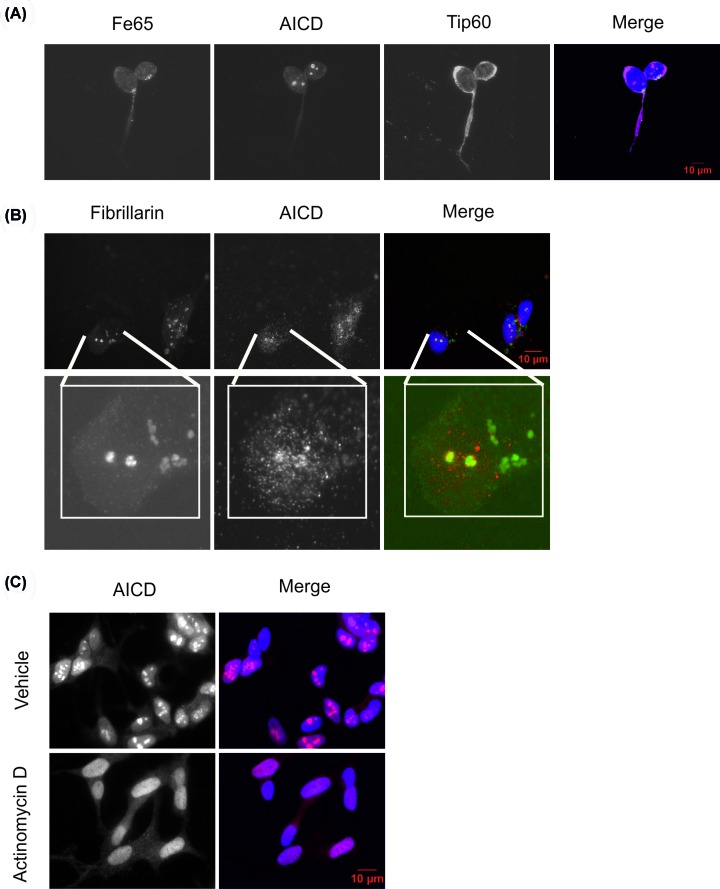
AICD is present in nucleoli SH-SY5Y cells were cultured and differentiated as described, followed by fixation and immunocytochemistry using primary antibodies against (**A**) FE65, AICD and Tip60 (**B**) AICD and fibrillarin. (**C**) Cells were treated with actinomycin D (0.02 µg/ml, 3 h) and immunocytochemistry performed to assess the effect on AICD localisation.

In order to investigate the identity of these AICD-positive, TDP-43-negative subnuclear structures, we assessed whether they represented the AFT (AICD-Fe65-Tip60) transcriptional regulatory complex. However, there was no evidence of Fe65 or Tip60 co-staining with these structures. Moreover, Tip60 immunostaining is absent from these AICD-positive puncta in a similar manner to TDP-43 (Figure 2A). It is unclear exactly how the endogenous AFT complex manifests. Several publications report the use of fluorescently tagged AFT complex components [48–51], but Tip60 in these studies shows a markedly different distribution to those reports using smaller tags, or untagged Tip60 [52–54]. Further differences may arise from the use of non-neuronal [51,52] or neuronal-like [53] cell lines. Altogether, these data give a contradictory picture of the exact nature of the endogenous AFT complex in neurons.

Given the large size of the AICD-positive structures, it was hypothesised that they may represent nucleoli. Indeed, the nucleolar marker fibrillarin showed strong co-localisation with AICD, indicating that AICD may be localised to the nucleolus (Figure 2B). Localisation of AICD to nucleoli has not previously been reported in the literature. Pharmacological treatment with actinomycin D is known to disrupt nucleoli and treatment of SH-SY5Y cells resulted in a homogenous redistribution of AICD throughout the nucleus (Figure 2C). Taken together, these data indicate that nuclear AICD may be localised to nucleoli, from which TDP-43 is excluded, potentially suggesting that there is no direct interaction between TDP-43 and APP/AICD.

### Modulation of APP expression does not affect TDP-43 expression

**Figure 3:**
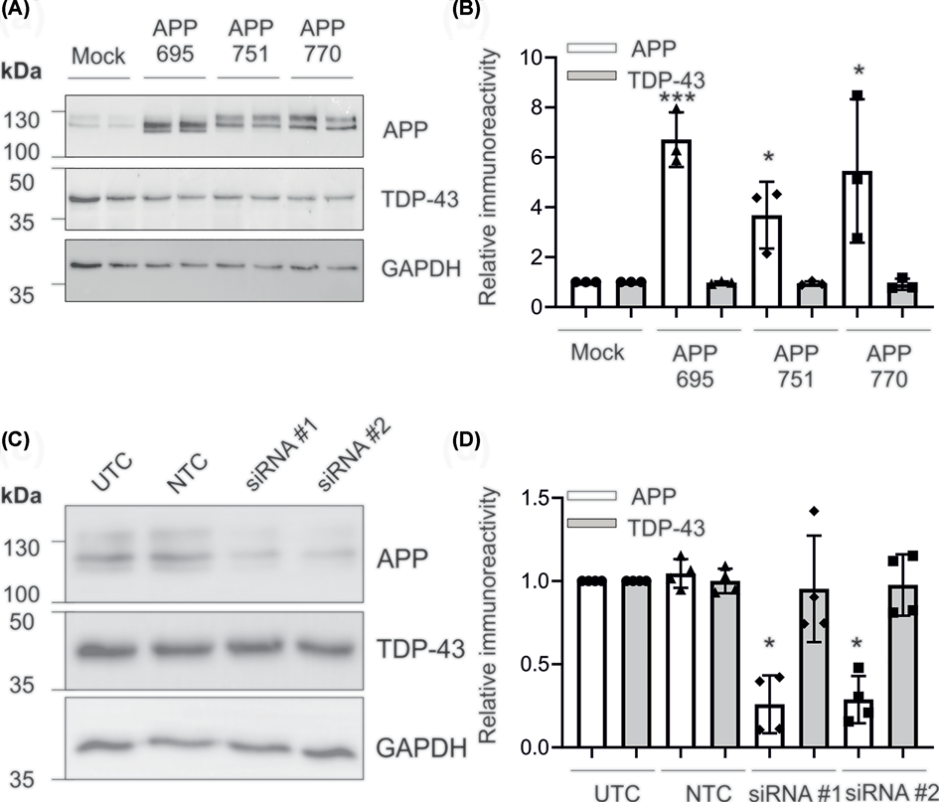
APP overexpression or knockdown does not affect TDP-43 expression SH-SY5Y cells were cultured as described and (**A**) stably transfected with each of the three APP isoforms. Expression was confirmed by immunoblot, compared with TDP-43 expression and (**B**) changes in protein expression quantified. (**C**) Cells were nucleofected with two independent siRNA targeting APP and protein expression of APP and TDP-43 assessed by immunoblot and (**D**) quantified; *n*=4. *P<0.05 and ***P<0.001.

APP has been shown to regulate the expression of numerous genes, both directly through AICD and indirectly via its binding partners [17,55,56]. In order to assess whether APP could modulate the expression of TDP-43, each APP isoform (695, 751, 770) was expressed separately in SH-SY5Y cells. Individual isoforms were expressed to assess any differential effects of APP, as AICD is preferentially generated from APP695, suggesting isoform-specific processing pathways [42]. There were no substantial isoform differences in the level of APP expression, with all isoforms expressed at an approximately eight-fold higher level than in the mock control (Figure 3A,B). None of the APP isoforms had an effect on TDP-43 immunoreactivity (Figure 3A,B). Similarly, siRNA-mediated knockdown of APP had no effect on TDP-43 expression (Figure 3C,D).

### Inducible expression of TDP-43 does not affect expression of proteins involved in APP proteolytic processing

**Figure 4:**
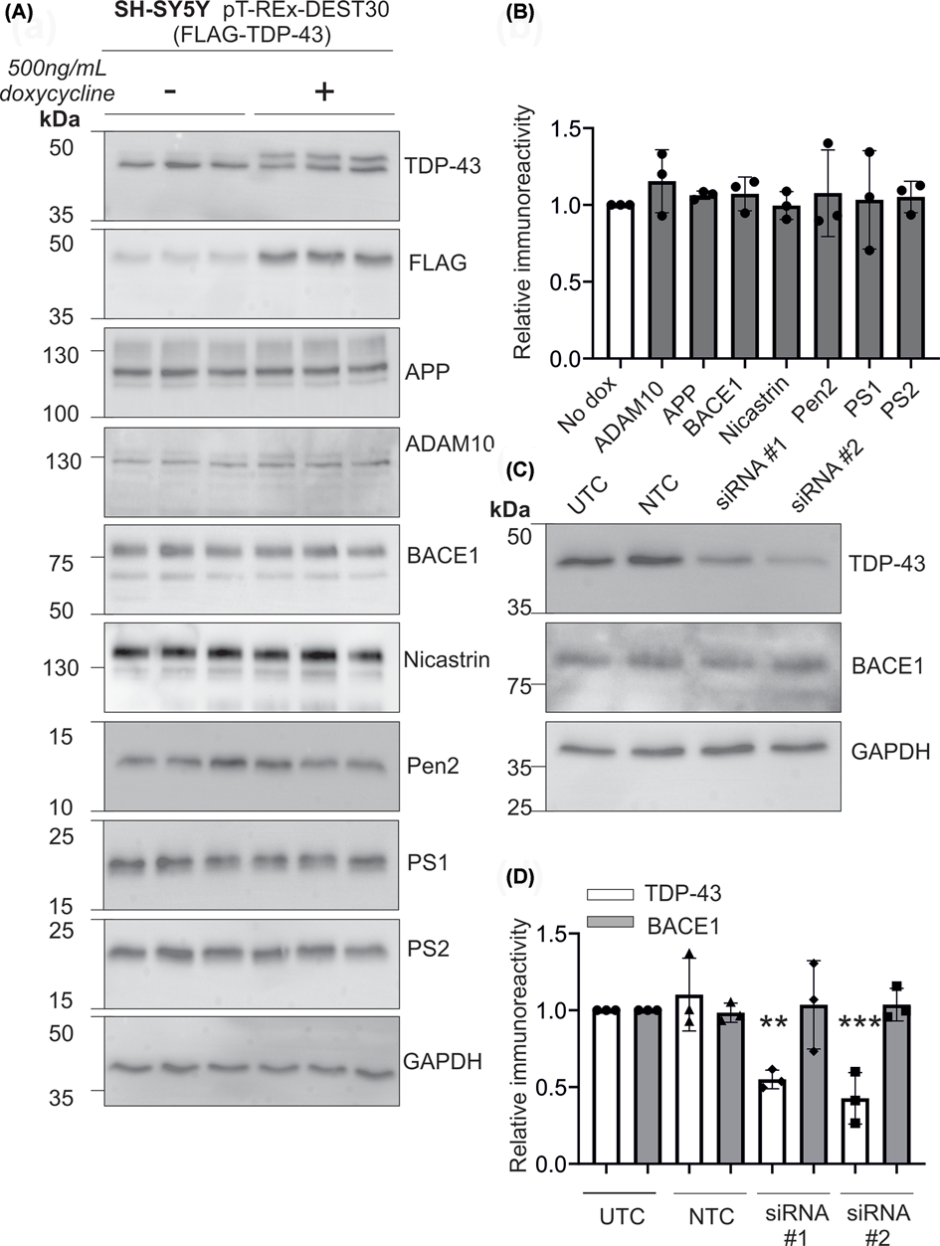
Inducible TDP-43 overexpression does not affect APP processing A stable cell line with doxycycline-inducible TDP-43 expression (pT-REx-DEST30-TDP-43-FLAG) was generated and (**A**) TDP-43 overexpression confirmed by immunoblot. APP and its proteolytic enzymes were also probed by immunoblot and (**B**) changes quantified by densitometry. (**C**) Cells were nucleofected with two independent siRNA targeting TDP-43 and protein expression of TDP-43 and BACE1 assessed by immunoblot and (**D**) quantified. **P<0.01 and ***P<0.001.

**Figure 5:**
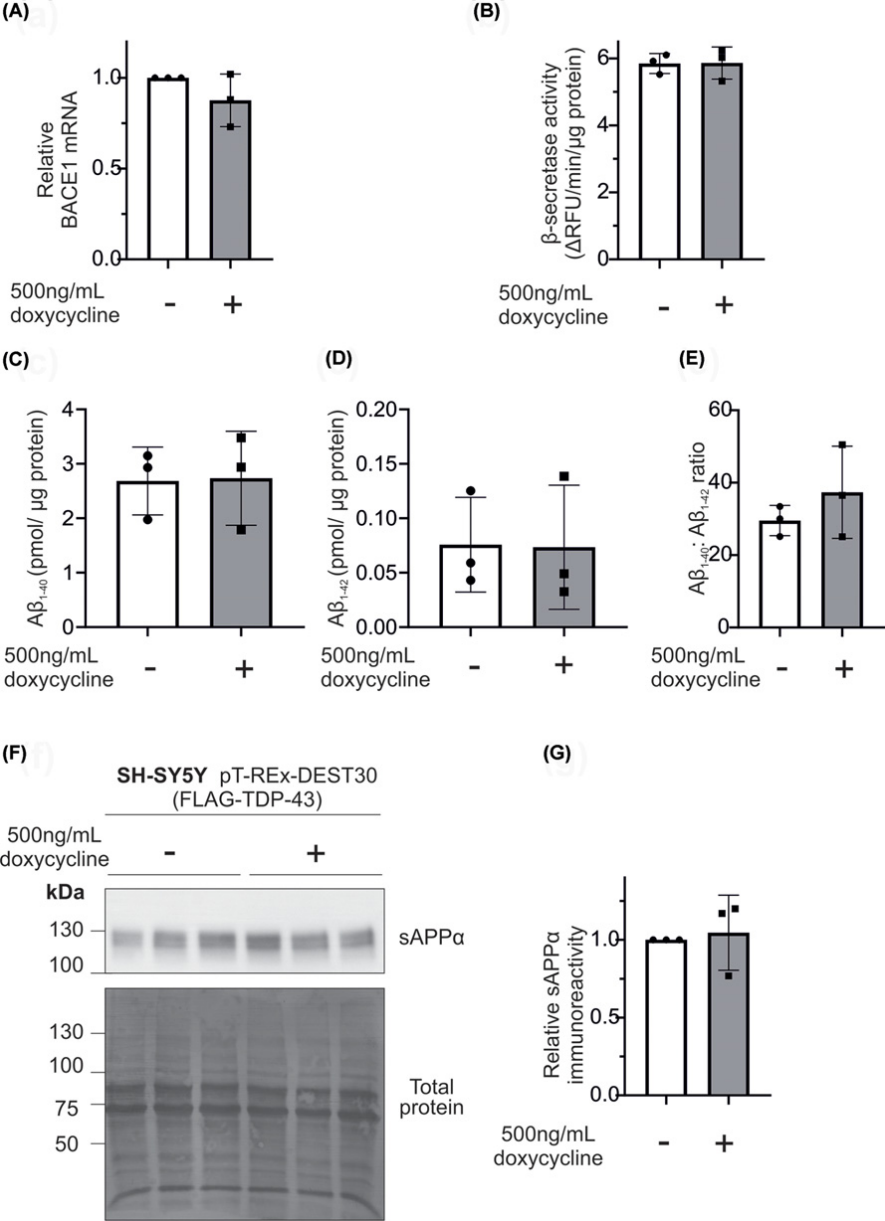
TDP-43 overexpression does not modulate BACE1 mRNA, activity or the generation of Aβ peptides TDP-43 expression was induced in SH-SY5Y (pT-REx-DEST30-TDP-43-FLAG) cells and cells were analysed for (**A**) BACE1 mRNA levels, (**B**) β-secretase activity and culture medium assessed for (**C**) Aβ1-40 and (**D**) Aβ1-42 followed by (**E**) calculation of the Aβ1-40: Aβ1-42 ratio (**F**) sAPPα immunoreactivity (using antibody 22C11). Total protein was assessed using Amido Black stain to ensure equal loading and (**G**) protein changes were quantified by densitometry.

An inducible cell line was generated, whereby TDP-43 was only expressed when activated by doxycycline. Incubation of cells with doxycycline resulted in increased expression of TDP-43, combined with FLAG immunoreactivity (Figure 4A), indicating high expression of FLAG-TDP-43 when induced with doxycycline. Using this cell model, TDP-43 expression was induced and the expression of APP and key APP-related proteins assessed. The protein panel comprised the APP holoprotein, the α-secretase ADAM10, the β-secretase BACE1 and the γ-secretase components (PS1, PS2, nicastrin and Pen2). Overall, the expression of TDP-43 did not have any significant effect on the expression of any of these proteins (Figure 4A,B). Given a previous report linking TDP-43 to BACE1 [41], we further focused on these proteins. Using siRNA knockdown, we assessed the effect of a reduction in TDP-43 on the expression of BACE1. Despite significant reductions in TDP-43 expression, this did not alter the immunoreactivity of BACE1 (Figure 4C,D). Our data show a lack of change in BACE1 protein expression in response to TDP-43 overexpression or knockdown. To confirm that TDP-43 did not alter BACE1 protein expression, qPCR was used to measure the level of BACE1 mRNA. After doxycycline-induced expression of TDP-43, there was no significant change in BACE1 mRNA (Figure 5A). Overall, these data show that TDP-43 does not affect the expression of key proteins involved in APP proteolytic processing.

### TDP-43 expression does not alter APP proteolysis or BACE1 activity

APP processing is an enzymatic process and, as such, may be modulated by changes in secretase activity which are independent of protein expression. In order to assess this, β-secretase activity was assessed using a fluorescent substrate. Doxycycline-induced expression of TDP-43 had no effect on β-secretase activity (Figure 5B). Increased expression of TDP-43 as induced by doxycycline did not have any effect on levels of Aβ_1-40_ or Aβ_1-42_, nor did it affect the ratio between the two peptides (Figure 5C–E). The mean abundance in the absence of doxycycline treatment was 2.68 pg/µg protein (Aβ_1-40_) or 0.076 pg/µg protein (Aβ_1-42_) and in the presence of doxycycline was 2.73 pg/µg protein (Aβ_1-40_) or 0.073 pg/µg protein (Aβ_1-42_). In addition, there was no effect on non-amyloidogenic processing of APP, as immunoblotting conditioned medium for sAPPα showed no change upon TDP-43 induction (Figure 5F,G). Together these data show that TDP-43 does not modulate either amyloidogenic or non-amyloidogenic proteolytic processing of APP. Our data conflicts with Herman et al. who reported TDP-43 mediated up-regulation of BACE1 in a mouse model [41]. However, their lentiviral overexpression of TDP-43 also caused significant increases in TNFα and IL-6, both of which can increase BACE1 expression and activity [41]. Due to the confounding inflammation, it is not possible to determine whether TDP-43 directly increases BACE1 activity or merely promotes an inflammatory phenotype which drives BACE1 activity. However, as we observed no direct effect of TDP-43 on BACE1 activity, it is more likely that the increased BACE1 activity in the mice was due to the inflammation.

## Conclusion

In the present study, we have focused on the interaction of TDP-43 with APP proteolytic processing. TDP-43 did not influence APP expression, nor did APP modulate TDP-43 expression. TDP-43 had no effect on the non-amyloidogenic cleavage of APP and did not alter the production of either Aβ_1-40_ or Aβ_1-42_. In addition, TDP-43 did not directly affect BACE1 activity. Our data strongly suggest that TDP-43 is not directly involved in any AD-linked alterations in APP expression or proteolytic cleavage. Given these findings and the notable regional differences between TDP-43 and Aβ pathology in AD [26], it is highly likely that AD-linked alterations in Aβ are independent of TDP-43 dysfunction.

## Abbreviations

AD: Alzheimer’s disease
ADAM10: a disintegrin and metalloprotease 10
AICD: amyloid precursor protein intracellular domain
APP: amyloid precursor protein
Aβ: amyloid β
BACE1: β-site APP cleaving enzyme 1
BCA: bicinchoninic acid
BSA: bovine serum albumin
ELISA: enzyme-linked immunosorbent assay
FBS: foetal bovine serum
FSG: fish skin gelatin
iPSC: induced pluripotent stem cell
iPSN: iPSC-derived neuron
PBS: phosphate-buffered saline
qPCR: quantitative PCR
sAPPα: soluble amyloid precursor protein α
SDHA: succinate dehydrogenase complex, subunit A
TBST: TBS + 1% (v/v) Tween-20
TDP-43: transactive response DNA binding protein 43
